# Germline variants in UNC13D and AP3B1 are enriched in COVID-19 patients experiencing severe cytokine storms

**DOI:** 10.1038/s41431-021-00886-x

**Published:** 2021-04-19

**Authors:** Hui Luo, Dan Liu, Wenbing Liu, Gaoxiang Wang, Liting Chen, Yang Cao, Jia Wei, Min Xiao, Xin Liu, Gang Huang, Wei Wang, Jianfeng Zhou, Qian-fei Wang

**Affiliations:** 1 Department of Hematology, Tongji Hospital, Tongji Medical College, Huazhong University of Science and Technology, Wuhan, Hubei China; 2 CAS Key Laboratory of Genomic and Precision Medicine, Beijing Institute of Genomics, Chinese Academy of Sciences, Beijing, China; 3 China National Center for Bioinformation, Beijing, China; 4 University of Chinese Academy of Sciences, Beijing, China; 5 Divisions of Pathology and Experimental Hematology and Cancer Biology, Cincinnati Children’s Hospital Medical Center, Cincinnati, OH USA; 6 Department of Neurology, Tongji Hospital, Tongji Medical College, Huazhong University of Science and Technology, Wuhan, Hubei China

**Keywords:** Immunogenetics, Viral infection

## Abstract

Critically ill coronavirus disease 2019 (COVID-19) is characterized by severe cytokine storms, a hyperinflammatory condition intimately related to the development of fatal outcomes. Why some individuals seem particularly vulnerable to severe cytokine storms is still unknown. Primary immunodeficiency (PID)-related genes are inherited factors that dysregulate host inflammatory responses to infection, especially hemophagocytic lymphohistiocytosis (HLH)-related genes, established as contributors to the development of excessive cytokine storms. We analyzed the association between PID gene variants with severe cytokine storms in COVID-19. We conducted whole-exome sequencing in 233 hospitalized COVID-19 patients and identified four PID gene (*UNC13D*, *AP3B1*, *RNF168*, *DHX58*) variants were significantly enriched in COVID-19 patients experiencing severe cytokine storms. The total percentage of COVID-19 patients with variants in *UNC13D* or *AP3B1*, two typical HLH genes, was dramatically higher in high-level cytokine group than in low-level group (33.3 vs. 5.7%, *P* < 0.001). Germline variants in *UNC13D* and *AP3B1* were associated with the development of severe cytokine storms, fatal outcomes in COVID-19. These findings advance the understanding of individual susceptibility to severe cytokine storms and help optimize the current management of COVID-19.

## Introduction

Coronavirus disease 2019 (COVID-19) is triggered by severe acute respiratory coronavirus 2 (SARS-CoV-2), taking on various degrees of severity, ranging from mild to critical illness [1]. Critically ill or intensive care unit (ICU)-admitted COVID-19 is characterized by severe cytokine storms, a hyperinflammatory condition intimately related to the development of lethal acute respiratory distress syndrome, multiple organ failure, and even death [2, 3]. One question about the cytokine storms still lingers: why some individuals seem particularly vulnerable? Few relevant research studies are now underway exploring the underlying genetic mechanisms.

Germline variants of primary immunodeficiency (PID)-related genes, including defects in innate and adaptive immunity, have been seen as major inherited factors that dysregulate host inflammatory responses to infection [4–6]. A set of 12 PID genes (*PRF1, UNC13D, STX11, STXBP2, RAB27A, LYST, AP3B1, SH2D1A, BIRC4, ITK, CD27*, and *MAGT1)* have been established as contributors to the development of cytokine storms, and are dubbed hemophagocytic lymphohistiocytosis (HLH)-related genes [7]. Despite their obvious clinical significance, the mutational status of PID genes, especially HLH genes has not been investigated in patients with COVID-19.

## Materials and methods

### Patients and sample collection

The study population consisted of 233 participants with newly diagnosed COVID-19 between January 13, 2020 and March 5, 2020 at Tongji Hospital, Huazhong University of Science & Technology, Wuhan, China. Informed consent was obtained from each patient or patients’ relatives. The detailed information was shown in Supplementary materials.

### High- and low-level cytokine groups

The levels of serum cytokines were determined by Bio-Plex Pro Human Cytokines 48-Plex Screening (Bio-Rad Life Sciences, Hercules, CA, USA) on a Luminex FlEXMAP 3D system (Luminex, Austin, TX, USA) by following the manufacturer’s protocols. The relative levels (fold change relative to healthy donors) of IP-10, IL-Ra, or MCP-3 were used to perform receiver operating characteristic (ROC) curve analysis to discriminate between ICU and non-ICU patients. ICU admission, as a status variable, was used in ROC analysis since COVID-19 patients admitted to ICU tended to experience severe cytokine storms.

### Exome sequencing and rare, damaging variants filtering

All coding variants were generated from whole-exome sequencing (WES) or targeted exome sequencing of DNA samples. We constructed a knowledge base of 241 genes involved in host antiviral immunity (Table S1). The rare variants (minor allele frequency (MAF) ≤ 0.01 in overall populations from public database 1000 G and gnomAD) were identified. The damaging variants meant having a functional impact of the proteins and were predicted by in silico analysis based on PolyPhen-2, SIFT, and CADD. Variants were classified as damaging by at least two software predicted as damaging. The analysis strategies in detail were shown in Supplementary materials.

### Sequencing data availability

The raw sequence data have been deposited in the Genome Sequence Archive [8] in NGDC [9], under accession number HRA000392. The variants data have been deposited in the LOVD database (https://databases.lovd.nl/) and GVM database [10] (https://bigd.big.ac.cn/gvm/getProjectDetail?project=GVM000127).

## Results

We performed WES on 233 hospitalized COVID-19 patients (mild, *n* = 58; severe, *n* = 133; critical, *n* = 42) with an attempt to understand the correlation between PID genes and severe cytokine storms in COVID-19. The median age was 60 years (interquartile range: 46–68 years). More clinical data are detailed in Table S2 and analysis strategies presented in Fig. S1.

**Fig. 1:**
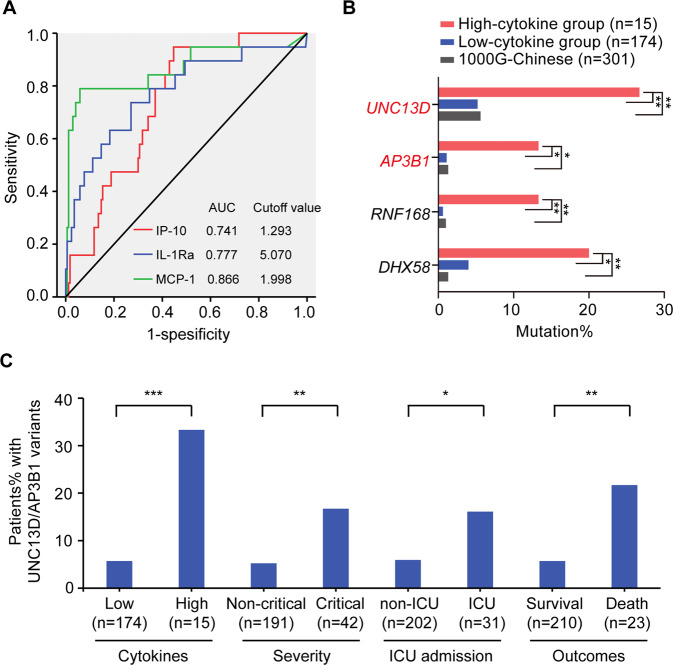
Germline variants were significantly enriched in COVID-19 patients with severe cytokine storms. **A** The red, blue, and green lines represent the receiver operating characteristic (ROC) curves of IP-10, IL-Ra, or MCP-3 to discriminate COVID-19 patients with ICU admission or without ICU admission, respectively. The areas under the curve (AUC) and the optimal cutoff values set by Youden’s index are shown in the graph. **B** The bar chart shows the mutation percentage of the four genes in high- and low-level cytokine groups and in Chinese population from 1000 G. The statistically significant mutation burden was analyzed by the chi-square test and the Fisher’s test. *P < 0.05; **P < 0.01. **C** The stacked bar shows the total percentage of patients harboring UNC13D or AP3B1 variants. The patients were grouped according to the severity of cytokine storms, COVID-19 severity, ICU admission, and fatal outcomes. The statistical significance between the groups was assessed by the chi-square test. *P < 0.05; **P < 0.01; ***P < 0.001.

The severity of cytokine storms in COVID-19 was rated in terms of three cytokines (IP-10, IL-1Ra, and MCP-3) as previously reported [11]. Patients were included in high-level cytokine group (*n* = 15) when all three cytokines were significantly increased (above the cutoff values), and the others were assigned to low-level cytokine group (*n* = 174), see Fig. 1A and Supplementary materials. There were no significant differences in the term of age, sex, comorbidity, duration of hospitalization between the high- and low-level cytokine groups, suggesting similar clinical baseline characteristics between the two groups (Table S3). We focused on PID genes by constructing a knowledge base of 241 antiviral immunity-related genes, which were mainly reported in PID diseases (see Supplementary materials and Table S1) [4]. We identified a total of 534 rare (MAF ≤ 0.01), damaging variants in 145 PID genes from 233 COVID-19 patients (listed in Table S4). To find out genes most relevant to the severe cytokine storms in COVID-19 patients, the variant burden of each identified genes was compared between high- and low- level cytokine groups. Variants in four genes were found to be significantly enriched in high-level cytokine group. UNC13D (26.7%, odds ratio (OR) = 6.7, 95% confidence interval (CI) = 1.8–25.1) was the most frequently mutated gene, followed by AP3B1 (13.3%, OR = 13.2, 95% CI = 1.7–101.7), RNF168 (13.3%, OR = 26.6, 95% CI = 2.3–313.3), and DHX58 (20.0%, OR = 6.0, 95% CI = 1.4–26.0). The variant burdens of these four genes were also strikingly higher in high-level cytokine group than in Chinese general population (*n* = 301) from the 1000 Genomes project (Fig. 1B and Table S5). UNC13D (encoding Munc13–4) and AP3B1 (encoding AP3), two typical HLH genes, were among the major players in the development of HLH due to defects in T/NK-cell cytotoxic granule release pathway. Three heterozygous missense variants (NM_199242.3:c.1607G > T:p.(Arg536Leu) [12], NM_199242.3:c.2588G > A:p.(Gly863Asp) [12, 13], and NM_199242.3:exon23:c.2242G > T:p.(Ala748Ser)) and one splice-site variant (NM_199242.3:exon17:c.1447-1G > A) in *UNC13D* and two heterozygous missense variants (NM_003664.5:exon23:c.2779G > A:p.(Gly927Ser), NM_003664.5:exon8:c.911 C > T:p.(Thr304Ile)) in *AP3B1* were found in high-level cytokine group. Among them, the NM_199242.3:c.2588G > A:p.(Gly863Asp) variant in *UNC13D* is predictably affected the MHD2 domain by the HOPE software. In high-level cytokine group, one patient had two variants in the *UNC13D* gene, and four patients had a monoallelic variant in the *UNC13D* or *AP3B1* gene. The total percentage of COVID-19 patients with variants in UNC13D or AP3B1 was dramatically higher in high-level cytokine group than in low-level group (33.3 vs. 5.7%, *P* < 0.001), in critical group than in non-critical group (16.7 vs. 5.2%, *P* < 0.01), in ICU than in non-ICU admission group (16.1 vs. 5.9%, *P* < 0.05), and in death group than in survival group (21.7 vs. 5.7%, *P* < 0.01) (Fig. 1C). To further validate our findings, we examined the variants in UNC13D and AP3B1 genes by targeted sequencing in an additional outpatient group including 51 asymptomatic SARS-CoV-2 infection individuals. Only 2.0 % (1 out of 51) of the individuals had the UNC13D variant, 3.9 % (2 out of 51) had AP3B1 variants (Table S6). The total percentage of patients with UNC13D and AP3B1 variant in the outpatient group was much lower than in high-level cytokine group (5.9 vs. 33.3%, *P* < 0.05) (Fig. S3).

## Discussion

Our study found that three types of PID gene variants were enriched in COVID-19 patients experiencing severe cytokine storms. UNC13D and AP3B1 variants might be associated with the development of severe cytokine storms, critical illness, and fatal outcomes in COVID-19 patients, while the functional relevance of two other type genes (antibody production gene RNF168 and viral sensor regulatory gene DHX58) warrants further investigation. A growing number of studies reported that heterozygous variants in HLH-related genes might be associated with HLH development [14–16]. Our study suggests that carrying a putatively deleterious variant in the UNC13D or AP3B1 gene might increase the risk of disease severity, possibly via interaction with other genetic mutations and/or nongenetic factors (e.g., exposure to SARS-CoV-2). Regarding the limitation of this study, we cannot differentiate functionally deleterious or neutral variants based on computational analysis. Except for the splicing site variant, all the other variants, including the in-frame variants with high CADD scores might be neutral when experimentally tested [17, 18] and the burden test should be further performed on experimentally proven deleterious variants. Our findings will be helpful in advancing the understanding of individual susceptibility to severe cytokine storms and optimizing the current management of COVID-19. For vulnerable populations genetically subject to the severe cytokine storms, more aggressive immunomodulation therapies should be considered [19].

## Supplementary information

Supplementary materials, Figure S1, Figure S2,Figure S3, Table S2, Table S3, Table S5

Supplementary Table S1, Table S4, Table S6, Table S7
