# Supplementary material for: Germline variants in UNC13D and AP3B1 are enriched in COVID-19 patients experiencing severe cytokine storms
Source: Eur J Hum Genet. 2021 Apr 19;29(8):1312–5. doi: 10.1038/s41431-021-00886-x (PMC8053565; doi:10.1038/s41431-021-00886-x)
Supplement: Supplementary file 1 — Supplementary materials, Figure S1, Figure S2,Figure S3, Table S2, Table S3, Table S5 [file 41431_2021_886_MOESM1_ESM.docx]

**SUPPLEMENTARY dATA**

**CONTENTS**

Materials and methods………………………………………………...………………1

Figure S1…………………………………………...………………………………… 9

Figure S2………………………………………...……………………………………10

Figure S4………………………………………………………………………………11

Table S2…………………………………………...…………………………………. 12

Table S3……………………………………………………………....…………..…...12

Table S5…………………………………………………………………………..…...13

References………………………………………………………..…………………..14

**MATERIALS AND METHODS**

**Patient selection**

The study population consisted of 233 participants with newly diagnosed COVID-19 between January 13, 2020 and March 5, 2020 at Tongji Hospital, Huazhong University of Science & Technology, Wuhan, China. This study was approved by the Medical ethics committee of Tongji Hospital, Tongji Medical College, Huazhong University of Science and Technology, Wuhan, China (TJ-C20200113). Written informed consent was obtained from 230 patients and waived in 3 patients who died and only oral consent was obtained from the relatives of the patients. We included the 3 patients in the study in light of the urgent pandemic as other published studies did.(1, 2) Written informed consent was also obtained from 51 outpatient cases for validation. Patients’ data were recorded in an electronic data capture system (EDCS), including demographics, medical history, oximetric assessment, swabbing results, radiography and clinical outcomes. Medical data from electronic health records were extracted by using a standardized data collection form. All patients enrolled were sporadic and had no family aggregation, and didn’t have family history of HLH or hyperinflammatory syndromes. Each medical ward used standardized guidelines to minimize variability in the diagnosis and estimation of the severity of COVID-19. Severity was classified on basis of the China National Health Commission Guidelines for 2019-nCoV infection (the 7th version) as previously reported.(3) COVID-19 patients with fever, respiratory symptoms and radiological findings of pneumonia were categorized as mild. COVID-19 patients who met one of the following criteria were considered as severe ones: (1) respiratory distress (respiration rate ≥ 30 times per minute); (2) resting oxygen saturation ≤ 93%; (3) arterial oxygen partial pressure/ fraction of inspired oxygen ≤ 300 mmHg. COVID-19 patients who satisfied one of the following criteria were taken as critical cases: (1) respiratory failure requiring mechanical ventilation, (2) shock, (3) failure of other organs requiring intensive care unit. The discharge criteria included clinical criteria (having no following manifestations: consciousness disorder, BUN > 7 mmol/L, respiratory rate ≥ 30 times/min, systolic pressure < 90 mmHg or diastolic pressure ≤ 60mmHg, PaO2/FiO2 ≤ 300 mmHg), imaging criteria (The lung lesion area reduced by more than 50%) and SARS-CoV-2 detection results [negative results of two consecutive nucleic acid swab tests (time interval ≥ 24h)]. Patients could be discharged when they met all aforementioned criteria. This study also included cryopreserved serum samples collected from 22 healthy donors for cytokine determination.

**Sample collection**

In this study, the serum samples were prospectively collected within the first 72 h of hospitalization. Blood samples were collected and immediately transferred to a 4℃ refrigerator and were processed within 24 hours. Serum samples were harvested using a serum separator tube (SST) and samples were allowed to coagulate for 30 minutes at room temperature before centrifugation for 15 minutes at 1000 **×** g. The serum was taken and assayed immediately or aliquoted, and samples were stored at ≤ -80 ℃. Repeated freeze-thaw cycles were avoided. Peripheral blood cells were collected into ethylene diamine tetraacetic acid (EDTA)-anticoagulant tubes and centrifuged for 15 minutes at 1000 **×** g. Peripheral blood cells were obtained after removing plasma and red blood cells by using red blood cell lysis buffer. Genomic DNA was extracted from peripheral blood cells with a QIAmp DNA Blood Mini kit (Qiagen, Germany) according to the manufacturer’s instructions.

**Measurement of cytokines**

The levels of serum cytokines were determined by Bio-Plex Pro Human Cytokines 48-Plex Screening (Bio-Rad Life Sciences, Hercules, CA, USA) on a Luminex FlEXMAP 3D system (Luminex, [Austin, TX](https://goo.gl/maps/kHcFpH6cn3k), USA) by following the manufacturer’s protocols. The 48-Plex Screening panel: Basic FGF, CTACK, eotaxin, G-CSF, GM-CSF, GRO-α, HGF, ICAM-1, IFN-α2, IFN-γ, IL-1α, IL-1Ra, IL-2, IL-2Ra, IL-3, IL-4, IL-5, IL-6, IL-7, IL-8, IL-9, IL-10, IL-12, IL-13, IL-15, IL-16, IL-17A, IL-18, IP-10, LIF, MCP-1, MCP-3, M-CSF, MIF, MIG, MIP-1α, MIP-1β, β-NGF, PDGF-BB, RANTES, SCF, SCGF-β, SDF-1α, TNF-α, TNF-β, TRAIL, VCAM-1, VEGF-A. Data were analyzed using Bio-Plex Manager 6.2 software package (Bio-Rad Life Sciences, Hercules, CA, USA). For non-appreciable levels, random values between 0 and the limit of detection (LOD) were inputted to avoid the artificial reduction of the standard deviation.

**High- and low-level cytokine groups**

The relative levels (fold change relative to healthy donors) of IP-10, IL-Ra or MCP-3 were used to perform receiver operating characteristic (ROC) curve analysis to discriminate between ICU and non-ICU patients. ICU admission, as a status variable, was used in ROC analysis since COVID-19 patients admitted to ICU tended to experience severe cytokine storms. The severity of cytokine storms in COVID-19 was evaluated in terms of three cytokines (IP-10, IL-1Ra and MCP-3) as previously reported.(3) The areas under the curve (AUC) was used to evaluate the three cytokines (IP-10, IL-1Ra, MCP-1) in distinguishing between ICU and non-ICU admission patients (AUC of IP-10, IL-1Ra and MCP-3 were 0.741, 0.777, 0.866, respectively). *Youden*’s index was used to find out the optimal cutoff values (The cutoff values of IP-10, IL-1Ra and MCP-3 were 1.293, 5.070, 1.998, respectively), which maximized the sensitivity and specificity to differentiate ICU and non-ICU admission patients.(4) Patients were assigned to a high-level cytokine group when the levels of IP-10, IL-1Ra, MCP-3 were simultaneously above the cutoffs, and the others were included in low-level cytokine group.

**Whole exome sequencing and targeted exome sequencing**

All coding variants were generated from whole exome sequencing (WES) of the genomic DNA samples. DNA fragments were end-repaired, ligated to the adapters, then amplified. Exome capture was performed using the BGI Human All Exon V5 kit, according to the manufacturer's instructions. All samples were sequenced using BGISEQ-2000 platform (BGI Corporation at Wuhan). We obtained an average of 109,441,084 reads from each sample. Targeted exome sequencing was performed by Ion Proton (Life Technologies) after multiplexing PCR by the Ion AmpliSeq Library kit 2.0 (Life Technologies) for HLH-related genes. The similar analytical process for WES was used to filter the predicted damaging variants in candidate genes.

**Sequence alignment and variants detection**

FASTQC tool ([*http://wwwbioinformaticsbabrahamacuk/projects/fastqc/*](http://wwwbioinformaticsbabrahamacuk/projects/fastqc/)) was used to assess the quality of raw sequence data (reads). Burrows-Wheeler Aligner (BWA) was used to map 2 x 150 pairs of end reads (Depth, 200x) to the human reference genome (build hg38).(5) Duplicate reads were removed using Picard software (<http://broadinstitute.github.io/picard/>). The Genome Analysis Tool kit (GATK version 3.8.0) was used to process the alignments, such as indel local realignment and base recalibration.(6) The processed alignment files (.bam) were further analyzed for germline variants calling. The GATK HaplotypeCaller was applied for calling single nucleotide variants and small insertions /deletions (Indels).(7) These variants were annotated using ANNOVAR software (version 2017-07-16)(8).

**Construction of a knowledge base of anti-viral immune genes**

We constructed a knowledge base of 241 genes involved in host anti-viral immunity (listed in supplementary Table S2). Of them, 190 genes were from the primary immunodeficiency (PID) diseases and 51 genes from other known anti-viral immune genes.(9) These genes could be divided into three major categories according to biological functions (Table S2): (1) Genes involved in pattern recognition receptors signaling pathways; (2) Genes related to the T/NK T-cells immune responses; (3) Genes associated with B cells activation and antibody secretion. The rare predicted damaging variants were filtered on the basis of the knowledge base.

**Filtering of rare, predicted damaging variants**

Among the defined germline variants that passed the quality control, we focused on rare variants with potential damaging effects. The synonymous variants were excluded. The rare variants [minor allele frequency (MAF) ≤ 0.01 in general overall populations] were identified. The 1000 Genome Project (1000G) and the genome Aggregation Database (gnomAD) were used to filter the rare variants. The damaging variants meant having a functional impact of the proteins and were predicted by *in silico* analysis based on three distinct algorithms. The three different algorithms were PolyPhen-2 (Polymorphism Phenotyping v2)(10), SIFT (Sorting Intolerant From Tolerant)(11) and CADD (Combined Annotation Dependent Depletion)(12). Variants were classified as predicted deleterious if at least two tools were predicted as damaging. Mutation Significance Cutoff (MSC) 99% CI were uses as the threshold(13). All variants meet the criteria as follow: HGMD-based MSC-PolyPhen2 99% prediction was “high”, HGMD-based MSC-SIFT 99% prediction was “high” or HGMD-based MSC-CADD 99% prediction was “high”.

**Gene variant burden tests**

The variant burden of each identified genes in high-level cytokine group was compared with that of low-level cytokine group and the general Chinese population (N = 301) from the 1000 Genomes project (http://ftp.1000genomes.ebi.ac.uk/vol1/ftp/release/20130502), by using the chi-square test and the Fisher’s test in R version 3.5.2 (<https://cran.r-project.org/web/packages/logistf/index.html>) . The burden test is based on presence or absence of rare predicted damaging variants in each candidate gene(14). Then, we compared the combined frequency of these variants in each gene in individuals between high- and low-level cytokine groups, or between high-level cytokine group and the 1000G-Chinese. Mutated genes with *P* < 0.05 were believed to be significant (supplementary table S4).

***Statistical analysis***

All statistical analyses were performed by using the SPSS, GraphPad Prism V5 and R3.5.2. P-value < 0.05 was considered to be statistically significant.

***Sequencing data availability***

The raw sequence data reported in this paper have been deposited in the Genome Sequence Archive (Genomics, Proteomics & Bioinformatics 2017) in National Genomics Data Center (Nucleic Acids Res 2020), Beijing Institute of Genomics (China National Center for Bioinformation), Chinese Academy of Sciences, under accession number HRA000392 that are publicly accessible at https://bigd.big.ac.cn/search/?dbId=hra&q=HRA000392. The variations data have been deposited in the Genome Variation Map in National Genomics Data Center, Beijing Institute of Genomics (China National Center for Bioinformation), Chinese Academy of Sciences, under accession number GVM000127 that are publicly accessible at https://bigd.big.ac.cn/gvm/getProjectDetail?project=GVM000127. The variations also uploaded to the Leiden Open-source Variation Database (LOVD) at <https://databases.lovd.nl/> with the individual ID #00334283-#00334327, #00334343-#00334355, #00334358-#00334371, #00334373-#00334378, #00334381, #00334387, #00334390, #00334393-#00334406, #00334477-#00334500, #00334506, #00334511, #00334513, #00334523-#00334526, #00334528-#00334530, #00334533, #00334535-#00334548, #00334842-#00334872, #00334874, #00334876, #00334879, #00334901, #00334903, #00334918, #00334920, #00334923, #00334924, #00334926, #00334929, #00334931, #00334934, #00334937, #00334939, #00334940- #00334942, #00334944, #00334946, #00335006, #00335008- #00335030, #00335032-#00335043.

**SUPPLEMENTARY FIGURES**


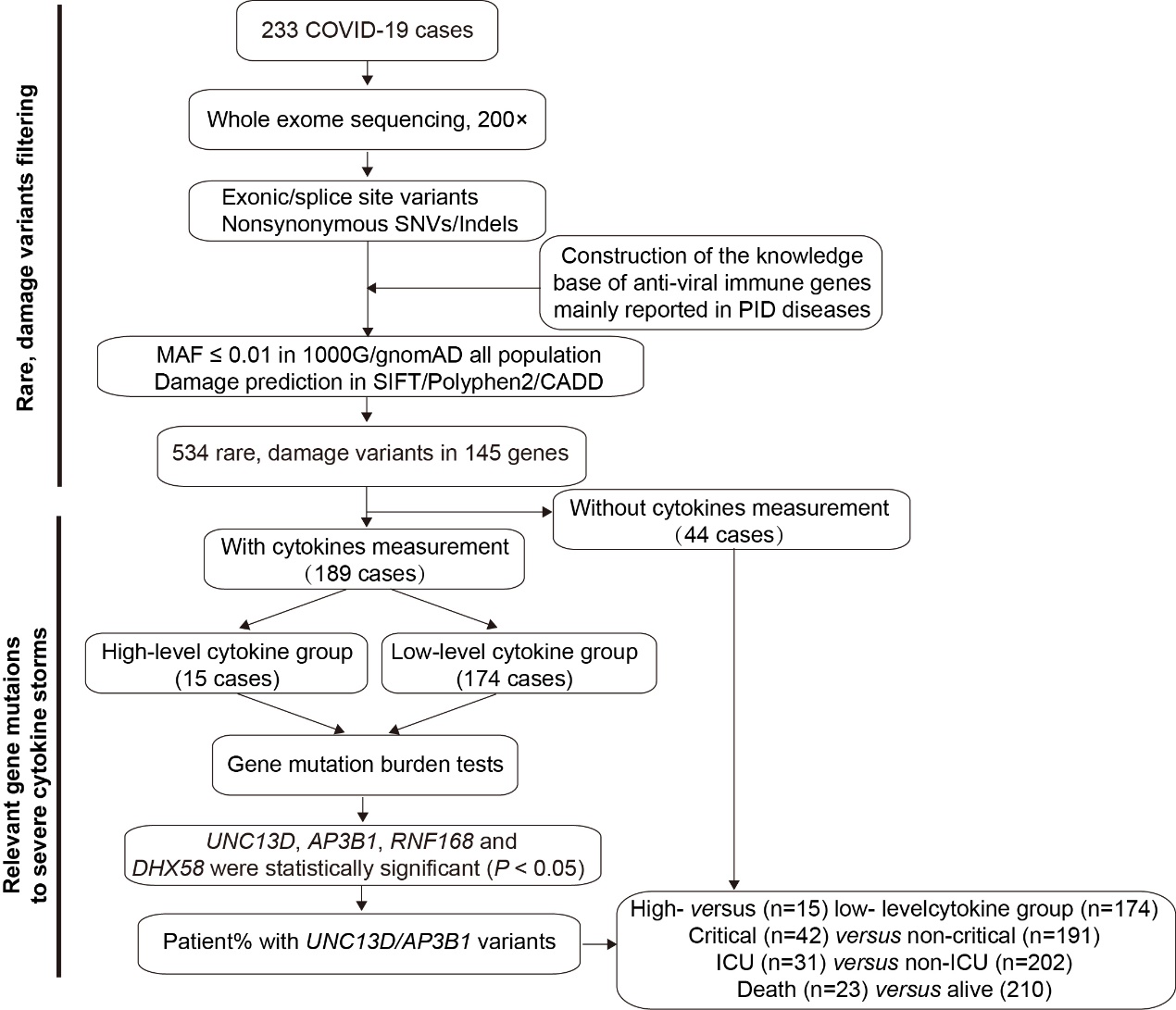


**Figure S1. Strategies for filtering rare predicted damaging variants and identifying gene variants associated with severe cytokine storms.** Strategies are detailed in supplementary materials and methods. SNV, single nucleotide variants; Indel, insertion deletion; PID, primary immunodeficiency; MAF, minor allele frequency; 1000G, the 1000 Genome Project; gnomAD, the Genome Aggregation Database; ICU, intensive care unit; SIFT, polyphen2 and CADD: variants damaging predicted software.


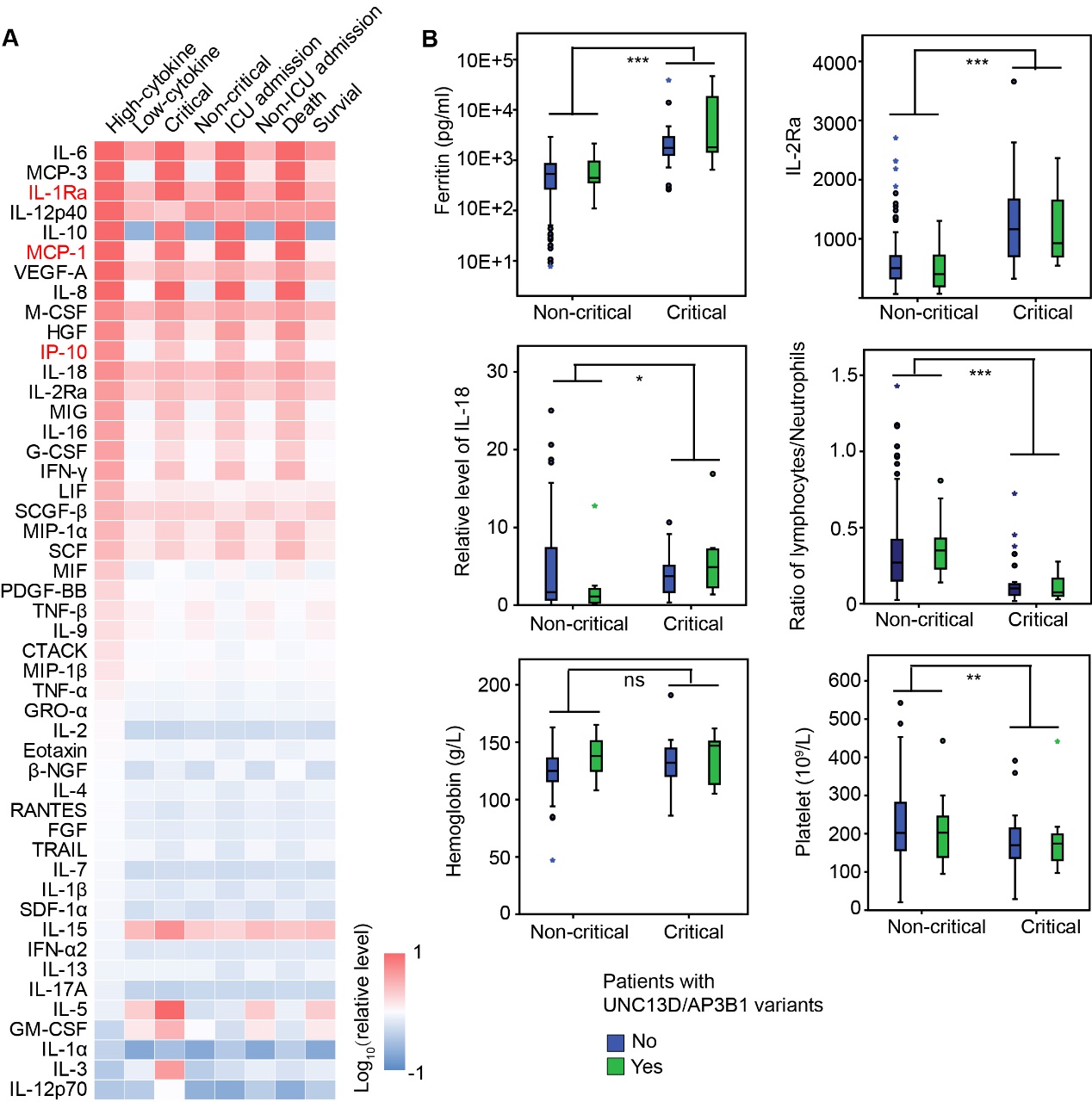


**Figure S2. The level of 48 cytokines and HLH-related laboratory results in different patient groups.** (A) The heatmap shows the average relative level of 48 cytokines in high- and low- level cytokine groups, critical and non-critical illness groups, ICU and non-ICU admission groups, death and survival groups. Many cytokines (IP-10, IL-1Ra, MCP-1, IFN-γ, IL-6, IL-8, IL-18, MCP-3, M-CSF, MIG, MIP-1α, SCF, IL-16, HGF, *etc*.) were significantly elevated in high-level cytokine group, critical illness group, ICU admission group and death group as compared with control groups. (B) HLH-related laboratory results (ferritin, IL-2Ra, IL-18, ratio of lymphocytes/ neutrophils, hemoglobin and platelet) in critical and non-critical illness COVID-19 with or without *UNC13D*/*AP3B1* variants are shown in the diagram. The level of ferritin, IL-2Ra and IL-18 in critical illness group was significantly higher than in non-critical group, while the ratio of lymphocytes/neutrophils and counts of platelet were significantly lower. There was no significant difference in the level of HLH-related laboratory results between patients with *UNC13D*/*AP3B1* variants and those without the variants. The statistical significance between the groups was assessed by Mann-Whitney tests. *, *P* < 0·05; **, *P* < 0·01; ***, *P* < 0·001; ns, not significant.


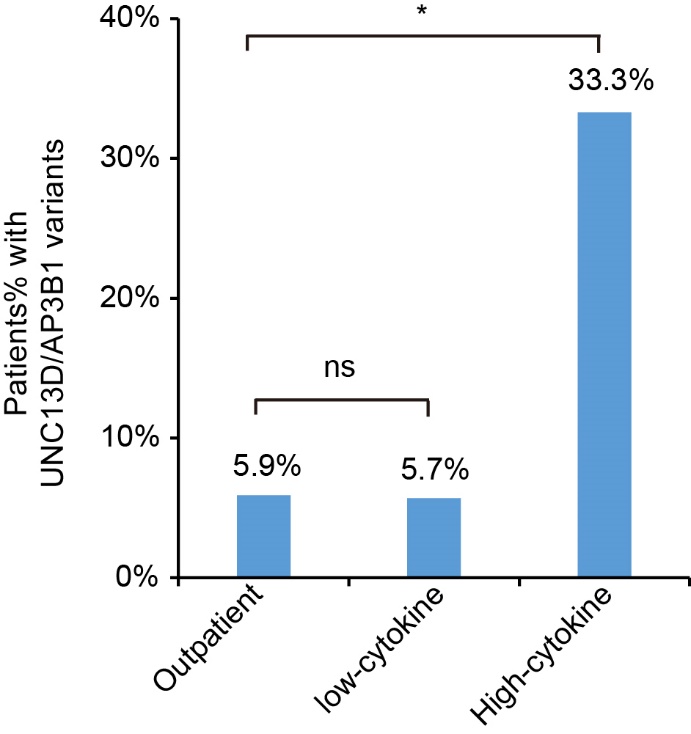


**Figure S3. Mutation burdens of *UNC13D* or *AP3B1* in different COVID-19 groups.** The bar chart shows the percentage of patients with *UNC13D*/*AP3B1* variants in high- and low-level cytokine groups and in an outpatient group including 51 asymptomatic carriers of SARS-CoV-2. *, *P* < 0.05; ns, not significant.

| **Table S2. Characteristics of 233 COVID-19 patients** | | | | | | | | |
| --- | --- | --- | --- | --- | --- | --- | --- | --- |
| **Characteristics** | | | **Non-critical (N=191)** | | **Critical (N=42)** | **Total (N=233)** | | |
| **Age, median (IQR), years** | | | 58 (44-57) | | 65 (58-72) | 60 (46-68) | | |
| **Sex, female, No. (%)** | | | 108 (56.5) | | 10 (23.8) | 118 (50.6) | | |
| **Comorbidity, No. (%)** | | |  | |  |  | | |
| Hypertension | | | 61 (31.9) | | 16 (38.1) | 77 (33.0) | | |
| Coronary artery heart disease | | | 9 (4.7) | | 7 (16.7) | 16 (6.9) | | |
| Diabetes | | | 28 (14.7) | | 8 (19.0) | 36 (15.5) | | |
| Severe obesity | | | 0 (0.0) | | 1 (2.4) | 1 (0.4) | | |
| Chronic lung disease | | | 1 (0.5) | | 2 (4.8) | 3 (1.3) | | |
| Chronic kidney disease | | | 1 (0.5) | | 1 (2.4) | 2 (0.9) | | |
| Cancer | | | 4 (2.1) | | 4 (9.5) | 8 (3.4) | | |
| **Highest level of respiratory support, No. (%)** | | |  | |  |  | | |
| Nasal cannula/facial mask oxygen therapy | | | 162 (84.8) | | 0 (0.0) | 162 (71.2) | | |
| Non-invasive mechanism ventilation | | | 0 (0.0) | | 19 (45.2) | 19 (8.2) | | |
| Invasive mechanism ventilation | | | 0 (0.0) | | 23 (54.8) | 23 (9.9) | | |
| **Treatment, No. (%)** | | |  | |  |  | | |
| Antibiotics | | | 133 (69.6) | | 38 (90.5) | 171 (73.4) | | |
| Antivirals | | | 171 (89.5) | | 37 (88.1) | 208 (89.3) | | |
| Corticosteroids | | | 94 (49.2) | | 35 (83.3) | 129 (55.4) | | |
| Intravenous immunoglobin | | | 30 (15.7) | | 14 (33.3) | 44 (18.9) | | |
| ECMO | | | 0 (0.0) | | 3 (7.1) | 3 (1.3) | | |
| Renal replacement therapy | | | 0 (0.0) | | 8 (19.0) | 8 (3.4) | | |
| **ICU care, No. (%)** | | | 0 (0.0) | | 31 (73.8) | 31 (13.3) | | |
| **Death, No. (%)** | | | 0 (0.0) | | 23 (54.8) | 23 (9.9) | | |
| **Duration of hospitalization, median days (IQR)** | | | 34 (28-44) | | 40 (29-51) | 36 (28-46) | | |
| No. (%): number (percentage of each group); IQR: interquartile range; ECMO: extracorporeal membrane oxygenation; ICU: intensive care unit. | | | | | | | | |
| **Table S3. Comparison of clinical characteristics between low-cytokine group and high-cytokine group.** | | | | | | |  |  |
| **Characteristics** | **Low-cytokine group (N=174)** | | **High-cytokine group (N=15)** | | | ***P*-value** |  |  |
| **Age, median (IQR), years** | 60 (43-68) | | 64 (57-70) | | | ns |  |  |
| **Sex, female, No. (%)** | 93 (53.4) | | 5 (33.3) | | | ns |  |  |
| **Comorbidity, No. (%)** |  | |  | | |  |  |  |
| Hypertension | 57 (32.8) | | 8 (53.3) | | | ns |  |  |
| Coronary artery heart disease | 12 (6.9) | | 3 (20.0) | | | ns |  |  |
| Diabetes | 25 (14.4) | | 3 (20.0) | | | ns |  |  |
| Chronic lung disease | 2 (1.1) | | 0 (0.0) | | | ns |  |  |
| Chronic kidney disease | 1 (0.6) | | 0 (0.0) | | | ns |  |  |
| Cancer | 5 (2.9) | | 1 (6.7) | | | ns |  |  |
| **Highest level of respiratory support, No. (%)** |  | |  | | |  |  |  |
| Nasal cannula/facial mask oxygen therapy | 131 (75.3) | | 2 (13.3) | | | <0.001 |  |  |
| Non-invasive mechanism ventilation | 12 (6.9) | | 2 (13.3) | | | ns |  |  |
| Invasive mechanism ventilation | 4 (2.3) | | 11 (73.3) | | | <0.001 |  |  |
| **Treatment, No. (%)** |  | |  | | |  |  |  |
| Antibiotics | 125 (71.8) | | 14 (93.3) | | | ns |  |  |
| Antivirals | 153 (87.9) | | 13 (86.7) | | | ns |  |  |
| Corticosteroids | 84 (48.3) | | 13 (86.7) | | | 0.004 |  |  |
| Intravenous immunoglobin | 29 (16.7) | | 7 (46.7) | | | 0.01 |  |  |
| ECMO | 0 (0.0) | | 3 (20.0) | | | <0.001 |  |  |
| Renal replacement therapy | 0 (0.0) | | 8 (53.3) | | | <0.001 |  |  |
| **ICU care, No. (%)** | 7 (4.0) | | 12 (80.0) | | | <0.001 |  |  |
| **Death, No. (%)** | 6 (3.4) | | 10 (66.7) | | | <0.001 |  |  |
| **Duration of hospitalization, median days (IQR)** | 34 (28-47) | | 36.5 (29.25-43.25) | | | ns |  |  |
| No. (%): number (percentage of each group); IQR: interquartile range; ECMO: extracorporeal membrane oxygenation; ICU: intensive care unit; ns, not significant. | | | | | | |  |  |

| **Table S5. The gene mutation burden in high- and low- level cytokine groups and in general Chinese populations** | | | | | | | | |
| --- | --- | --- | --- | --- | --- | --- | --- | --- |
| Gene | High-level cytokine group | Low-level cytokine group | *P*- value (Low- cytokine *versus* High-cytokine) | | 1000G-Chinese | | *P*- value (1000G-Chinese *versus* High-cytokine) | |
|  | (N=15) | (N=174) | Fisher exact test | Chi-square test | (N=301) | Fisher exact test | | Chi-square test |
| ***UNC13D*** | **4 (26.7)** | **9 (5.2)** | **0.012** | **0.009** | **17 (5.6)** | **0.012** | | **0.008** |
| ***AP3B1*** | **2 (13.3)** | **2 (1.1)** | **0.032** | **0.027** | **4 (1.3)** | **0.028** | | **0.018** |
| ***RNF168*** | **2 (13.3)** | **1 (0.6)** | **0.017** | **0.007** | **3 (1.0)** | **0.019** | | **0.007** |
| ***DHX58*** | **3 (20.0)** | **7 (4.0)** | **0.035** | **0.04** | **4 (1.3)** | **0.003** | | **0.000** |
| No. (%): number of patients (percentage in each group); 1000G, the 1000 Genomes project; *P* < 0.05, statistically significant. | | | | | | | | |

**REFERENCES**

1. Guan WJ, Ni ZY, Hu Y, Liang WH, Ou CQ, He JX, et al. Clinical Characteristics of Coronavirus Disease 2019 in China. The New England journal of medicine. 2020;382(18):1708-20.

2. Huang C, Wang Y, Li X, Ren L, Zhao J, Hu Y, et al. Clinical features of patients infected with 2019 novel coronavirus in Wuhan, China. Lancet (London, England). 2020;395(10223):497-506.

3. Yang Y, Shen C, Li J, Yuan J, Wei J, Huang F, et al. Plasma IP-10 and MCP-3 levels are highly associated with disease severity and predict the progression of COVID-19. The Journal of allergy and clinical immunology. 2020.

4. Le CT. A solution for the most basic optimization problem associated with an ROC curve. Statistical methods in medical research. 2006;15(6):571-84.

5. Li H, Durbin R. Fast and accurate short read alignment with Burrows-Wheeler transform. Bioinformatics. 2009;25(14):1754-60.

6. McKenna A, Hanna M, Banks E, Sivachenko A, Cibulskis K, Kernytsky A, et al. The Genome Analysis Toolkit: a MapReduce framework for analyzing next-generation DNA sequencing data. Genome research. 2010;20(9):1297-303.

7. DePristo MA, Banks E, Poplin R, Garimella KV, Maguire JR, Hartl C, et al. A framework for variation discovery and genotyping using next-generation DNA sequencing data. Nature genetics. 2011;43(5):491-8.

8. Wang K, Li M, Hakonarson H. ANNOVAR: functional annotation of genetic variants from high-throughput sequencing data. Nucleic acids research. 2010;38(16):e164.

9. Picard C, Al-Herz W, Bousfiha A, Casanova JL, Chatila T, Conley ME, et al. Primary Immunodeficiency Diseases: an Update on the Classification from the International Union of Immunological Societies Expert Committee for Primary Immunodeficiency 2015. Journal of clinical immunology. 2015;35(8):696-726.

10. Adzhubei IA, Schmidt S, Peshkin L, Ramensky VE, Gerasimova A, Bork P, et al. A method and server for predicting damaging missense mutations. Nature methods. 2010;7(4):248-9.

11. Vaser R, Adusumalli S, Leng SN, Sikic M, Ng PC. SIFT missense predictions for genomes. Nature protocols. 2016;11(1):1-9.

12. Kircher M, Witten DM, Jain P, O'Roak BJ, Cooper GM, Shendure J. A general framework for estimating the relative pathogenicity of human genetic variants. Nature genetics. 2014;46(3):310-5.

13. Itan Y, Shang L, Boisson B, Ciancanelli MJ, Markle JG, Martinez-Barricarte R, et al. The mutation significance cutoff: gene-level thresholds for variant predictions. Nature methods. 2016;13(2):109-10.

14. Wang XJ, Lian TY, Jiang X, Liu SF, Li SQ, Jiang R, et al. Germline BMP9 mutation causes idiopathic pulmonary arterial hypertension. The European respiratory journal. 2019;53(3).
